# Current Knowledge on the Bioavailability of Thymol as a Feed Additive in Humans and Animals with a Focus on Rabbit Metabolic Processes

**DOI:** 10.3390/ani12091131

**Published:** 2022-04-28

**Authors:** Iveta Placha, Kristina Bacova, Lukas Plachy

**Affiliations:** 1 Centre of Biosciences of the Slovak Academy of Sciences, Institute of Animal Physiology, Soltesovej 4-6, 040 01 Kosice, Slovakia; bacovak@saske.sk; 2 University of Veterinary Medicine and Pharmacy, Komenskeho 73, 041 81 Kosice, Slovakia; 3 1st Department of Cardiology, East Slovak Institute of Cardiovascular Diseases, Ondavska 8, 040 11 Kosice, Slovakia; lukas.plachy@upjs.sk; 4 Faculty of Medicine, Pavol Jozef Safarik University in Kosice, Trieda SNP 457/1, 040 11 Kosice, Slovakia

**Keywords:** thymol, biological activity, health, human, animal

## Abstract

**Simple Summary:**

This review provides general information on the possible health benefits in animals and humans of herbal additives, particularly thymol, whose phenolic group is responsible for the neutralisation of free radicals, and information concerning its detection through body action, bioavailability and mechanisms in rabbits. Plants containing thymol have been used in traditional medicine for the treatment of various diseases, such as cardiovascular diseases, cancer and diabetes. Although a great number of in vitro studies of cardiovascular and cancer diseases are available, in vivo studies that confirm these findings have not been sufficiently reported. To determine the beneficial dose, further clinical studies are necessary, with preclinical comprehensive research on animal models.

**Abstract:**

The aim of this review is to describe the therapeutic effect of thymol on various human diseases, followed by its bioavailability in humans and animals. Based on our knowledge from the current literature, after thymol addition, thymol metabolites—mostly thymol sulphate and glucuronide—are detected in the plasma and urine of humans and in the plasma, intestinal content, faeces and tissues in rats, pigs, chickens, horses and rabbits after enzymatic cleavage. In rabbits, thymol absorption from the gastrointestinal tract, its distribution within the organism, its accumulation in tissues and its excretion from the organism have been described in detail. It is necessary and important for these studies to suggest the appropriate dose needed to achieve the required health benefits not only for animals but also for humans. Information from this review concerning the mode of action of thymol in animal organisms could also be applied to human medicine and may help in the utilisation of herbal medicine in humans and in veterinary healthcare. This review summarises the important aspects of thymol’s effects on health and its bioavailability in organisms, particularly in rabbits. In future, herbal-based drugs must be extensively investigated in terms of their mode of action, efficiency of administration and clinical effect.

## 1. Introduction

For many years now, the positive effects of aromatic plants and herbs in the treatment of various diseases have been well documented. Many effective and conventional drugs originate from plants, and, historically, they represent the first pharmacological compounds used in the treatment of many diseases [1,2,3]. Scientists have focused their attention on the composition of herbal extracts, the detection of their active compounds and their mode of action to ensure an effective therapeutic and nutritional dosage [4]. To understand the mechanism of action of these compounds and to determine their interaction with food and drugs, their absorption, distribution, metabolism and excretion all need to be investigated [5,6].

Currently, the widespread antibiotic resistance presents a global health problem, and it has forced medical researchers to return to the pre-antibiotic era [7,8]. For this reason, the development of new drugs of natural origin presents a big challenge for scientists [9,10]. More than half of the drugs approved by Food and Drug Administration (FDA) are composed of natural compounds from medicinal plants [11]. About 50% of modern drugs, and more than 60% of anti-cancer drugs, are developed from phytochemicals [12]. According to Chen et al. [13] and Newman et al. [14], plants and their natural compounds have been used as sources of antibiotics, analgesics, cardioprotective and other drugs for more than 5000 years. Currently, collaboration between the World Health Organization (WHO), the FDA, the European Medicines Agency (EMA) and the pharmaceutical industry is necessary in order to utilise the vast potential of traditional medicine for the development of herbal drugs for the treatment of different diseases [15]. Despite the fact that aromatic plants and herbs are considered natural and safer than synthetic drugs, they may present a risk of serious adverse effects [16]. For this reason, researchers have suggested that food and drug interactions must be extensively evaluated in terms of their absorption, excretion, distribution and metabolism [5,6].

Many gastrointestinal and respiratory diseases in pigs, poultry and young cattle [17,18,19] and mastitis in adult cattle [20] are very often treated with antibiotics [21]. Bacteria that are spread into the environment by the excrement of farm animals carry antimicrobial resistance genes [22], and they are directly, or by food consumption, transmitted to humans [23,24]. The preventive use of medicinal plants may help to lower the use of antibiotics for the treatment of farm animals [25]. However, this statement is not mentioned in any national strategy on antibiotic resistance [26]. In recent decades, veterinarians and farmers have shown increased interest in using herbal medicine with the goal of reducing the use of antibiotics [27]. In order to explore strategies to minimise the development of antimicrobial resistance, more clinical veterinary studies using medicinal plants are required [21]. 

In recent years, plant secondary metabolites and plant-derived extracts have received extensive attention. Thymol, a major compound of thyme (*Thymus vulgaris* L.), exhibits antioxidant properties related to its phenolic structure, which adsorbs and neutralises free radicals. Several preclinical studies have well documented the pharmacological properties of thymol on animal models, but thymol also exhibits multi-pharmacological properties against various human diseases [28,29,30,31]. 

Thymol has been evaluated in many studies, which confirmed its therapeutic uses for the treatment of disorders affecting the cardiovascular, nervous, respiratory and digestive systems [32,33,34,35]. 

WHO reported, that cardiovascular diseases accounted for the highest number of deaths in 2019, and they are expected to reach 22.2 million deaths in 2030 [36]. For this reason, finding safe, non-toxic and effective drugs for prevention of these diseases is a hot topic of research. As natural candidates, some medicinal plants could meet the requirements for such pharmacological effects [37]. The active components of medicinal plants, due to their chemical structure and ability to reduce and scavenge the production of free radicals, improve the activity of antioxidant enzymes and are able to downregulate the oxidative stress of the organism [38]. In the opinion of Chang et al. [37], natural medicinal plants could be less toxic than synthetic antioxidants. Accumulating evidence suggests that flavonoids, phenolics and saponin from medical plants could reduce oxidative stress [16]. Moreover, some phenolic phytochemicals have anti-carcinogenic and anti-inflammatory effects [37,39]. Local inflammation and oxidative stress are involved in the pathogenesis of endothelial dysfunction and, consequently, in the progression of atherosclerosis [30]. Oxidative stress, by way of regulating inflammation and stimulating vascular smooth muscle, plays a key role in the pathogenesis of hypertension, atherosclerosis and myocardial injury [37, 40,41,42]. Oxidative stress represents an imbalance between free radical formation and antioxidant defence. An excessive amount of free radicals attack lipids in cells and cause their dysfunction, which induces the process of atherogenesis [43].

De Brito Alves et al. [44] suppose that the dietary intake of phenolic compounds could prevent hypertension through the beneficial impact on the gut microbiome. As much as 90–95% of phenolic compounds are accumulated in the large intestine, where they are converted into active metabolites by gut microbiota and, in turn, can modulate gut microbial composition by supporting the growth of certain beneficial bacteria [45,46]. Natarajan et al. [47] suggested that short-chain fatty acids, the major final product of faecal bacterial activity, could be responsible for lowering blood pressure. However, future studies are necessary to find a relationship between plant-derived phenolic compounds and the gut microbiome in connection with the prevention of hypertension [44,48]. In order to test the possible mechanism of action of these compounds, it would be interesting to design in vitro culture models that simulate intestinal tract conditions or to find a suitable animal model [30, 49]. Yu et al. [30] showed that rabbits, in comparison with other laboratory animals, are the most suitable model for the study of atherosclerosis in humans due to their similar lipoprotein metabolism.

The strong antimicrobial properties of thymol have been suggested by several studies. Thymol can be useful in the therapy of intestinal problems and respiratory infections [50]. Based on the studies of Komaki et al. [51] and Asadbegi et al. [52], the extract of *T. vulgaris* is considered as neuroprotective and is used to prevent anxiety. Thymol’s phenolic structure neutralises free radicals and exhibits redox properties [30]. In addition to in vitro and in vivo studies, clinical studies are required to establish the most effective way and dosage regimen of thymol administration [50].

To find the therapeutically relevant effects of plant compounds, reliable pharmacokinetic studies in humans are necessary [53]. An appropriate dose that would ensure the expected effect, absorption and excretion of phenolic compounds after thyme extract intake was studied in humans by Rubió et al. [54] and Kohlert et al. [55]. These data may also be important in the context of the safe application of herbal medicinal products [53]. However, relatively few studies on the bioavailability and pharmacokinetics of thymol, particularly in humans, are available to date [55]. To monitor phenolic consumption, hydroxytyrosol metabolites were analysed as a biomarker. However, the results showed its low specificity [54]. The authors showed that the metabolites of thymol, thymol sulphate and glucuronide were more specific, and they established the relationship between exposure and effect more precisely.

Present studies do not only focus their attention on the chemical composition and pharmacology of medicinal plants; their main objectives are the study of metabolites, the products of cell metabolism and their mechanism of action [56]. In order to propose the optimal dosage regimen, it is necessary to take into account that the metabolites of plant compounds could probably be deconjugated to the parental compounds and express their pharmacological activity in this way [54, 55]. Thymol sulphate and thymol glucuronide are the main metabolites of thymol, but, unfortunately, there is no information whether these compounds are active or inactive forms [50]. Thyme has often been used in folk medicine, and, recently, scientists have focused their attention on the pharmacodynamic activities of compounds in vitro, as well as their bioavailability in target organs [55].

The present review focuses in greater detail on the application of thymol as a natural feed additive in humans and animals to improve their health, with the aim being to substitute synthetic drugs. The limited information available on the bioactivity of thymol and its metabolites in organisms suggested to us to summarise the current knowledge of its absorption, distribution, accumulation and excretion. In the following sections, we try to compile all of the available literature in order to provide information concerning the tissues in which thymol and its metabolites have been detected to date and to describe the processes of its biotransformation, absorption, distribution, deposition and elimination and bioavailability in human and animal organisms.

## 2. Detection of Thymol and Its Metabolites in Humans and Animals

The pharmacodynamic activities of thyme extract or essential oil have been demonstrated in vitro. To confirm the beneficial effect of thymol found in vitro, its absorption, distribution, metabolism and excretion need to be detected in vivo [55]. To the best of our knowledge, thymol distribution in tissues in vivo has only been detected in more recent studies by several authors. To better understand the bioavailability of thymol in animal organisms and to establish the suitable concentration for beneficial effects on animal health, its metabolic path needs to be understood at the molecular level [57,58]. Little is known about the bioactivity of thymol and its metabolites, as there are only few studies that have analysed thymol in body tissues (Table 1).

According to our knowledge, the first study dealing with the metabolic fate of thymol, particularly its metabolite thymol glucuronide in the urine of rabbits and humans, was studied by Takada et al. [59]. The next early study was the examination of the glucuronide and sulphate conjugates of thymol as well as its isomer carvacrol in rat urine by Austgulen et al. [60]. Kraus and Ternes [61] detected thymol in egg yolk but not in albumin after the addition of thyme extract to laying hens. The transition of thymol from food into the yolk represented 0.006%.

The bioavailability of thymol as the main compound of thyme essential oil after the oral administration of a single dose of Bronchipret^®^ tablets (equivalent to 1.08 mg thymol) to humans was examined by Kohlert et al. [53]. No thymol was detected in plasma and urine; however, its metabolites thymol sulphate and glucuronide were found and excreted in urine, and sulphate was also detected in plasma. Thalhamer et al. [62] detected metabolite p-cymene-2,5-diol and its oxidised form (p-cymene-2,5-dione) as the main products after a single oral administration of 50 mg of thymol to humans. The authors showed that human metabolism leads to the preferred hydroxylation of the aromatic system of thymol. In comparison with the results of Austgulen et al. [60], they found that some of the metabolites that were found in rat metabolism (p-cymene-3,9-diol and p-cymene-3,7-diol) could not be detected in human samples and vice versa (p-cymene-2,3-diol and p-cymene-3,8-diol detected in human samples). For this reason, future studies of thymol metabolism in different species of vertebrates are necessary [63]. Michiels et al. [64] demonstrated that the absorption of thymol in piglets was intensive in the stomach and the proximal segments of the small intestine, and they also found rapid renal excretion of free and conjugated thymol. With the detected percentage of intake recovered in bile determined to be 4.1%, they showed that enterohepatic recycling cannot be neglected. Thymol concentrations have also been detected in the plasma, milk, liver, kidney and fat of cattle after intramammary administration of a herbal product containing the essential oil of *Thymus vulgaris* [65,66]. According to the studies of Kohlert et al. [55], Rubió et al. [67] and Rubió et al. [68], the free form of thymol could not be detected in plasma, but thymol conjugates originating from biotransformation in humans and animals were detected. Thymol glucuronide was found for the first time in rat plasma after a single oral dose of 1.5 g of thyme extract to animals, containing 44.32 µM of thymol [68]. The presence of thymol in blood plasma after enzymatic cleavage of the conjugates was detected in the plasma of horses after the administration of Bronchipret^®^ TP tablets with a thymol content of about 15 mg (the thymol concentration in plasma ranged from 62.4 to 315.9 ng/mL) by Van den Hoven et al. [69]. Haselmeyer et al. [70] detected thymol in broiler chickens fed with thyme herb (thymol concentrations in feed were 5–55 mg/kg). They found that thymol was quantified in the small intestine (115.5–1289.5 ng/g DM), the caecum (101.2–663.1 ng/g DM) and plasma (47.3–412.2 ng/mL), and it was only detected at the highest doses following enzymatic cleavage in the muscle and liver. However, the levels detected were relatively low. Haselmeyer [71] investigated the absorption of different thymol concentrations (2, 10 and 20 ug/mL) through the small intestine mucosa using the ex vivo method and Ussing chambers. He found that thymol concentrations in the mucosa/submucosa reached 7.5, 9.9 and 8.45% from the initial concentrations. Zitterl-Eglseer et al. [72] fed piglets with Biomin^®^ P.E.P 1000 feed additive (essential oil blended from oregano, anise and citrus peels; the main compounds thymol and carvacrol) and detected thymol at a low concentration in plasma (15.4 ng/mL), kidney (23.4 ng/g) and faeces (24.4 ng/g). In the study conducted by Hagmuller et al. [73], *Thymi herba* was given as a feed additive to weanling piglets at different concentrations (0.1, 0.5 and 1%). Thymol was detectable in all plasma samples, and the thymol level increased with greater amounts of thyme herbs. Fernandez et al. [74] validated for the first time an analytical extraction procedure (a headspace solid-phase microextraction technique followed by gas chromatography/mass spectrometry) as a method to detect and quantify thymol in the faeces and egg yolk of Japanese quail.

The thymol concentrations in the plasma, duodenal wall, liver, kidney, breast muscle and content of all the intestinal segments of broiler chickens were analysed after 4 weeks of consumption of thyme essential oil by Oceľová [63] and Oceľová et al. [75]. Thymol was determined through the length of the intestinal tract for the first time in these studies, and the results obtained pointed to intensive thymol absorption in the proximal part of the small intestine. They detected 37% of the thymol duodenal content in the duodenal wall, which confirmed its efficient absorption from the intestinal tract. The authors pointed to intensive metabolism in the liver and the subsequent transport of thymol to the kidneys, as the thymol concentration detected in the liver was 11 times lower than in the kidney. The lowest concentration of thymol was detected in muscle tissue. Pisarčíková et al. [76] focused their attention on detecting the two main thymol metabolites of the second phase of biotransformation processes, thymol sulphate and glucuronide in plasma, in the duodenal wall and liver. Thymol sulphate was detected at all levels of thyme essential oil addition (0.01, 0.05 and 0.1%), and the highest concentration was found in the duodenal wall, with the lowest in the liver. Thymol glucuronide was only detected in all biological samples at the highest thyme essential oil concentration (0.1%).

The small number of these studies indicates that there is insufficient information about thymol distribution in animal and human tissues despite the fact that the beneficial effect of thymol depends on its absorption and accumulation in the organism. In this section, we summarised the current knowledge of thymol and its metabolite deposition in different tissues.

**Table 1:** Detection of thymol and its metabolites in the bodies of humans and animals.

Animal Species	Applied Form	Detectable Compounds	Samples	References
human	thymol/orally	thymol glucuronide thymol sulphate thymohydroquinone sulphate thymol	urine	[59]
human	Bronchipret^®^ TP/orally (equivalent to 1.08 mg thymol)	thymol sulphate	plasma, urine	[55]
		thymol glucuronide	urine	
human	thymol/orally	p-cymene-2,5-diol p-cymene-2,3-diol p-cymene-3-ol-8-ene	urine	[62]
human	dried thyme/orally	thymol sulphate caffeic acid sulphate hydroxyphenylpropionic acid sulphate	plasma	[67]
		thymol sulphate caffeic acid sulphate hydroxyphenylpropionic acid sulphate thymol glucuronide	urine	
rabbit	thymol/orally	glucuronic acid ethereal sulphuric acid thymol	urine	[59]
rabbit	thymol/orally	thymol	plasma, small intestinal wall, liver, kidney, spleen, caecum, colon, muscle, faeces	[58]
rat	thymol/orally	p-cymene-2,5-diol p-cymene-3,9-diol p-cymene-3,7-diol thymol	urine	[60]
rat	thyme extract/orally	thymol sulphate	plasma	[68]
laying hen	thyme extract/orally	p-cymene-2,3-diol thymol	egg yolk	[61]
Japanese quail	thymol/orally	thymol	egg yolk, faeces	[74]
broiler chicken	dried *Thymi herba*/orally	thymol	plasma, small intestine, caecum, liver, muscle	[70]
broiler chicken	thyme essential oil/orally	thymol	plasma, liver, kidney, muscle, duodenal wall, gut content	[63]
broiler chicken	thyme essential oil/orally	thymol sulphate thymol glucuronide	plasma, duodenal wall, liver	[76]
piglet	essential oil/orally (carvacrol, thymol, eugenol and trans-cinnamaldehyde)	carvacrol thymol eugenol	plasma	[64]
		carvacrol thymol eugenol trans-cinnamaldehyde	small intestine	
		carvacrol thymol eugenol trans-cinnamaldehyde	bile	
		carvacrol thymol eugenol	urine	
piglet	Biomin^®^ P.E.P 1000 (main compounds thymol and carvacrol)/orally	thymol	plasma, kidney, faeces	[72]
piglet	*Thymi herba*/orally	thymol	plasma	[73]
bovine	Phyto-Mast (essential oil of *Thymus vulgaris* and oregano)/intramammary	thymol	milk, plasma, liver, kidney, fat	[65]
dairy cattle	Phyto-Mast (essential oil of *Thymus vulgaris* and oregano)/intramammary	thymol	milk, plasma, liver, kidney, fat	[66]
horse	Bronchipret (equivalent to 2–4 g thyme extract)/orally	thymol	plasma	[69]

## 3. Bioavailability of Thymol Generally and in Rabbits as Model Animal

To describe the metabolic processes of thymol in an organism, we chose the rabbit as a model animal because it represents an appropriate model for the evaluation of the bioavailability of nutrients. Phytogenic compounds and other foreign substances after oral administration are absorbed from the intestine, metabolised and eliminated from the organism. Once they reach the intestine or liver, they are converted during biotransformation processes (phase I and phase II) to more hydrophilic forms, and their pharmacological properties usually differ compared to the parental compound. It is also important to emphasise that metabolites can probably be deconjugated to the parental compound and express their pharmacological activity in this way. The major reactions occurring during phase I are oxidation, reduction and hydrolysis; phase II reactions are also called conjugation reactions and include glucuronidation, sulphation, acetylation, methylation, conjugation with glutathione and conjugation with amino acids [77,78,79,80,81,82].

The intestine plays an important role as a site for the absorption as well as biotransformation of thymol [63,76]. Thymol or its metabolites, after biotransformation processes in the intestinal wall, can be transported back into the intestinal lumen or are converted back to the parental compounds and redistributed within the organism through the systemic circulation [78]. Some part of the compounds are transported by the mesenteric vein into the liver, where they are metabolised, excreted into the duodenum in bile and again reabsorbed [57,78]. Bacova et al. [58] found 15% of thymol in the liver compared with the intestinal wall, which demonstrates the intensive absorption of thymol from the intestinal wall through the vena portae to the liver.

Oceľová [63] and Bacova et al. [58] detected significantly higher levels of thymol in the kidney in comparison with the liver and plasma, which shows that, although the liver is the most important organ where biotransformation processes occur, kidney microsomes are probably more effective. Kohlert et al. [55] showed that thymol metabolites are able to be reabsorbed in the proximal part of the kidney tubule, are cleaved by enzymes located in the brush border and are again reabsorbed.

There are some barriers that the compounds must pass through during their metabolic path in the organism, and they limit their absorption [63, 79]. The first-pass metabolism in the intestine decreases the number of molecules reaching the blood circulation, and then the first-pass metabolism in the liver represents another barrier for the distribution of compounds in the organism. In addition to this, many efflux transporters are bound mainly with lipophilic molecules, which are rapidly excreted from the organism, greatly limiting their bioavailability [58,78,80,81,82]. These biotransformation processes are probably the reason why only a trace amount of thymol is found in the muscle and fat tissues of broilers [70,77] and rabbits [58].

The rabbit’s digestive processes represent a complex system of the separation of digestible and indigestible parts of ingested food in the proximal colon [83]. The most important mechanism by which nutrients are released from ingested food is microbial fermentation in the caecum. The products of fermentation are either absorbed directly through the caecal wall or are re-ingested as caecotrophs [84]. The caecum and colon are the most important parts of a rabbit’s digestive system in connection with the original feature of digestion, caecotrophy. Bacova et al. [57], Bacova et al. [58] and Placha et al. [85], after thymol addition to a rabbit’s diet for 21 days and after its withdrawal from feed for the next 7 days, found a 6 times (7 times after withdrawal) higher concentration in plasma than in the intestinal wall. The authors attribute this finding to the metabolic processes that are present mainly in the caecum and that are responsible for the amount of thymol in caecotrophs. They stated that the mucus that covers the caecotrophs protects them, that the processes of fermentation continue until they reach the intestine and that thymol could be released and again reabsorbed into systemic circulation. The authors showed that, even though thymol was withdrawn, the metabolic processes of thymol were active, which was probably the consequence of caecotrophy.

Studying thymol bioavailability in different tissues is essential to understand its mechanism of action in target organs in which it can exert its biological role. Oral bioavailability represents the fraction of administered thymol reaching the systemic circulation and is a key parameter that affects its efficacy. Therefore, to propose an appropriate dose, the study of its oral bioavailability has received significant attention. However, only a few studies on thymol oral bioavailability have been carried out to date.

**Figure 1:**
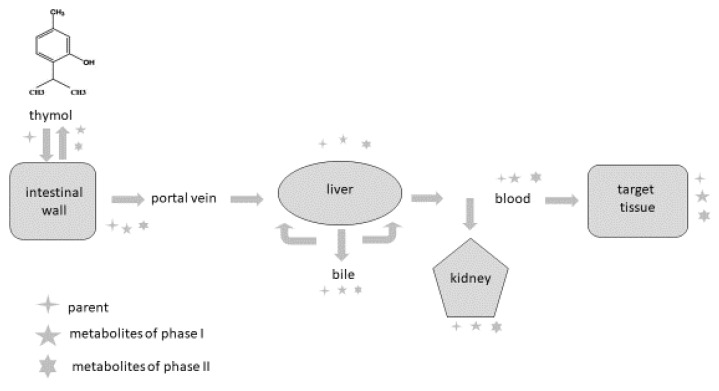
Organ bioavailability barriers to thymol (based on [79]).

The organ bioavailability barriers to thymol are depicted in Figure 1.

## 4. Conclusions

In conclusion, this literature review reports the future perspectives of modern thymol application in humans and animals. This review summarises the available literature on thymol application as a feed additive in humans and animals, with emphasis on its metabolic route and oral bioavailability. The presented data represent the available scientific information regarding the urgent need for more studies to precisely understand the metabolic processes and biological activity of thymol and its metabolites within organisms. This information will be useful for researchers, drug and pharmaceutical industries and the medical and veterinary sectors.

## Data Availability

The study did not report any data.
